# Supplementary material for: A UMLS-based spell checker for natural language processing in vaccine safety
Source: BMC Med Inform Decis Mak. 2007 Feb 12;7:3. doi: 10.1186/1472-6947-7-3 (PMC1805499; doi:10.1186/1472-6947-7-3)
Supplement: Additional file 13 — RAP application directory. Sets up directory for RAP (RDF application for PHP) source code [file 1472-6947-7-3-S13.gz › rap/doc/phpdoc/__filesource/fsource_syntax__syntaxN3Serializer.php.html]

xml version="1.0" encoding="iso-8859-1"?


File Source for N3Serializer.php


# Source for file N3Serializer.php

Documentation is available at N3Serializer.php

```
1. <?php

3. // ----------------------------------------------------------------------------------
4. // Class: N3Serializer
5. // ----------------------------------------------------------------------------------


9. /**
10. * PHP Notation3 Serializer
11. *
12. * This class serialises models to N3 Syntax.
13. *
14. * Supported N3 features:
15. * <ul>
16. *   <li>Using [ ] for blank nodes, or _: if nescessary</li>
17. *   <li>Literal datytype- and xmlLanguageTag support</li>
18. * </ul>
19. *
20. * Un-supported N3 Features include:
21. * <ul>
22. *   <li>Reification</li>
23. * </ul>
24. *
25. * <b>History:</b>
26. * <ul>
27. * <LI>12-06-2004 improved namespace handling added (tobias.gauss@web.de)</LI>
28. * <li>25-03-2004 Bug in doResource() fixed</li>
29. * <li>11-27-2003 fixed problems with detection for using [ ] or _: for blank nodes.
30. *                Added constant N3SER_BNODE_SHORT to disable the automatic detection  if bnode is referenced elsewhere</li>
31. * <li>11-19-2003 Support for datatypes and language tags added.</li>
32. * <li>08-01-2003 Made compatible with new v6 MemModel</li>
33. * <li>31-07-2003 Function saveAs added. <radol@gmx.de></li>
34. * <li>07-16-2003 Initial version</li>
35. * </ul>
36. *
37. * TODO: * added namespace prefixes are persisent...
38. *
39. * @author Gunnar AA. Grimnes <ggrimnes@csd.abdn.ac.uk>, Daniel Westphal <mail@d-westphal.de>
40. * @version V0.9.1
41. * @package syntax
42. * @access public
43. ***/

45. define('MAGIC_STRING', '~~~');

47. class N3Serializer extends Object {

49. var $debug;

51. var $prefixes;

53. var $done;  // keeps track of already serialized resources

55. var $resourcetext;
56. var $resourcetext_taken;
57. var $model;
58. var $res;
59. var $anon;

61. /**
62. * Constructor
63. *
64. * @access   public
65. */
66. function N3Serializer() {
67. $this->debug=FALSE;
68. }


71. /**
72. * Adds a new namespace prefix to use.
73. * Unknown namespaces will become ns0, ns1 etc.
74. * @access public
75. * @param string $s
76. * @returns void
77. ***/

79. function addNSPrefix( $ns, $prefix) {
80. $this->prefixes[$ns]=$prefix;
81. }

83. /**
84. * Serializes a model to N3 syntax.
85. *
86. * @param     object Model $model
87. * @return    string
88. * @access    public
89. */
90. function & serialize(&$m) {

92. if (is_a($m, 'DbModel'))
93. $m=$m->getMemModel();

95. $this->reset();
96. $this->model=$m;
97. $this->res="";

99. // copy default namespaces
100. global $default_prefixes;
101. foreach($default_prefixes as $prefix => $namespace)
102. $this->addNSPrefix($namespace,$prefix);

104. $nps= $this->model->getParsedNamespaces();
105. if($nps!=false){
106. foreach($nps as $uri => $prefix){
107. $this->addNSPrefix($uri,$prefix);
108. }
109. }

111. $namespaces=array();
112. $count=array();
113. $resources=array();
114. foreach ($this->model->triples as $t) {
115. $s=$t->getSubject();
116. if ( is_a($s, 'Resource'))
117. $namespaces[$s->getNamespace()]=1;

119. $p=$t->getPredicate();
120. if ( is_a($p, 'Resource'))
121. $namespaces[$p->getNamespace()]=1;

123. $o=$t->getObject();
124. if ( is_a($o, 'Resource'))
125. $namespaces[$o->getNamespace()]=1;

127. $uri=$s->getURI();

129. if (isset($count[$uri])) {
130. $count[$uri]++;
131. } else {
132. $count[$uri]=0;
133. $resources[$uri]=$s;
134. }
135. }

137. if (!HIDE_ADVERTISE)
138. $this->res .= '# Generated by N3Serializer.php from RDF RAP.'.LINEFEED
139. .'# http://www.wiwiss.fu-berlin.de/suhl/bizer/rdfapi/index.html'
140. .LINEFEED.LINEFEED;

142. $this->doNamespaces($namespaces);

144. $this->res.=LINEFEED.LINEFEED;

146. arsort($count);

148. foreach ( $count as $k=>$v) {
149. $this->doResource($resources[$k]);
150. //      $this->res.=" .\n";
151. }

153. $c=0;
154. foreach ( $this->resourcetext as $r=>$t) {

156. if ( preg_match_all('/'.MAGIC_STRING.'([^ ]+)'.MAGIC_STRING.'/', $t, $ms, PREG_SET_ORDER)) {

158. foreach($ms as $mseach) {
159. $rp=$this->resourcetext[$mseach[1]];
160. $t=preg_replace('/'.MAGIC_STRING.$mseach[1].MAGIC_STRING.'/', $rp, $t);
161. }
162. }

164. if ($this->debug) $this->res.=$c.': ';
165. if ( !( isset($this->resourcetext_taken[$r]) && $this->resourcetext_taken[$r]>0) )
166. $this->res.=$t.' .'.LINEFEED;
167. else if ( $this->debug )
168. $this->res.=' Skipping : '.$t.LINEFEED;
169. $c++;
170. }

172. //     $max=-1111;
173. //     $maxkey="";
174. //     foreach ($count as $k=>$c) {
175. //       if ( $c>$max) { $maxkey=$k; $max=$c; }
176. //     }

178. //     if ($this->debug) {
179. //       print "$maxkey is subject of most triples! ($max) \n";
180. //     }

182. return $this->res;
183. }

185. /**
186. * Serializes a model and saves it into a file.
187. * Returns FALSE if the model couldn't be saved to the file.
188. *
189. * @access    public
190. * @param     object MemModel $model
191. * @param     string $filename
192. * @return    boolean
193. * @access    public
194. */
195. function  saveAs(&$model, $filename) {

197. // serialize model
198. $n3 = $this->serialize($model);

200. // write serialized model to file
201. $file_handle = @fopen($filename, 'w');
202. if ($file_handle) {
203. fwrite($file_handle, $n3);
204. fclose($file_handle);
205. return TRUE;
206. }else{
207. return FALSE;
208. };
209. }


212. /* ==================== Private Methods from here ==================== */


215. /**
216. * Readies this object for serializing another model
217. * @access private
218. * @param void
219. * @returns void
220. ***/
221. function reset() {
222. $this->anon=0;
223. $this->done=array();
224. $this->resourcetext_taken=array();
225. $this->resourcetext=array();
226. $this->res='';
227. $this->model=NULL;
228. unset($this->prefixes);
229. }

231. /**
232. * Makes ns0, ns1 etc. prefixes for unknown prefixes.
233. * Outputs @prefix lines.
234. * @access private
235. * @param array $n
236. * @returns void
237. ***/
238. function doNamespaces(&$n) {
239. $c=0;
240. foreach ($n as $ns => $nonsense) {
241. if ( !$ns ) continue;
242. if ( isset($this->prefixes[$ns]) ) {
243. $p=$this->prefixes[$ns];
244. } else {
245. $p='ns'.$c;
246. $this->prefixes[$ns]=$p;
247. $c++;
248. }
249. $this->res.="@prefix $p: <".$ns.'> .'.LINEFEED;
250. }
251. }

253. /**
254. * Fill in $resourcetext for a single resource.
255. * Will recurse into Objects of triples, but should never look ? (really?)
256. * @access private
257. * @param object Resource $r
258. * @returns boolean
259. ***/

261. function doResource(&$r) {
262. //    print $r->getURI();

264. $ts=$this->model->find($r, null, null);
265. if (count($ts->triples)==0) return;

267. $out="";

269. if ( isset($this->done[$r->getURI()]) && $this->done[$r->getURI()] ) {
270. if ( is_a($r, 'BlankNode')) {

272. if ( $this->resourcetext_taken[$r->getURI()] == 1) {
273. //Oh bother, we must use the _:blah construct.
274. $a=$this->resourcetext[$r->getURI()];
275. $this->resourcetext[$r->getURI()]='_:anon'.$this->anon;
276. $this->resourcetext['_:anon'.$this->anon]=$this->fixAnon($a, '_:anon'.$this->anon);
277. $this->resourcetext_taken[$r->getURI()]=2;
278. $this->anon++;
279. }

281. }
282. return false;
283. }

285. $this->done[$r->getURI()]=TRUE;

287. if ( is_a($r, 'Resource') ) {

289. if ( is_a($r, 'BlankNode') ) {

291. //test, if this blanknode is referenced somewhere
292. $rbn=$this->model->find(null, null, $r);

294. if (count($rbn->triples)>0 | !N3SER_BNODE_SHORT) {
295. $out.='_:'.$r->getLabel();
296. } else {
297. $out.='[ ';
298. };
299. } else {

301. $this->doURI($r, $out);

303. };
304. };

306. usort($ts->triples, 'statementsorter');
307. $lastp='';

309. $out.=' ';

311. foreach ($ts->triples as $t) {

313. $p=$t->getPredicate();

315. if ($p == $lastp) {
316. $out.=' , ';
317. } else {
318. if ($lastp!='') $out.=' ; ';
319. $this->doURI($p, $out);
320. $lastp=$p;
321. }

323. $out.=' ';

325. $o=$t->getObject();

327. if ( is_a($o, 'Literal')) {
328. $l=$o->getLabel();
329. if ( strpos($l, LINEFEED) === FALSE ) {
330. $out.="\"$l\"";
331. } else {
332. $out.="\"\"\"$l\"\"\"";
333. }
334. if ( $o->getLanguage()!='' ) {
335. $out.='@'.$o->getLanguage();
336. }
337. if ( $o->getDatatype()!='' ) {
338. $out.='^^<'.$o->getDatatype().'>';
339. }

341. }

343. if (is_a($o, 'Resource')) {
344. if ($this->debug) print 'Doing object: '.$o->getURI().LINEFEED;
345. if ( is_a($o,'BlankNode')) {

347. //    $this->doResource($o);
348. //      $out.=MAGIC_STRING.$o->getURI().MAGIC_STRING; #$this->resourcetext[$o->getURI()];
349. //      $this->resourcetext_taken[$o->getURI()]=1;

351. $out .='_:'.$o->getLabel();
352. } else {

354. $this->doURI($o, $out);
355. }
356. }
357. }

359. if (isset($rbn) && !count($rbn->triples)>0 && N3SER_BNODE_SHORT) {$out.=' ] ';};
360. $this->resourcetext[$r->getURI()]=$out;

362. return TRUE;
363. }

365. /**
366. * Format a single URI
367. * @param string $s
368. * @access private
369. * @return void
370. ***/
371. function doURI(&$r, &$out) {
372. if ( $r->getURI()=='http://www.w3.org/1999/02/22-rdf-syntax-ns#type') {
373. $out.='a';
374. return;
375. }
376. if ($r->getNamespace()!='') {
377. $out.=$this->prefixes[$r->getNamespace()].':'.$r->getLocalName();
378. } else {
379. //Will this ever happen?
380. $out.=$r->getURI();
381. }
382. }


385. /**
386. * Fix the resourcetext for a blanknode where the _: construct was used
387. * @param string $s
388. * @param string $a
389. * @access private
390. * @return void
391. ***/
392. function fixAnon($t,$a) {
393. $t=preg_replace("/( \] $|^\[ )/", '', $t);

395. return $a.$t;
396. }

398. }

400. ?>
```

Documentation generated on Thu, 7 Jul 2005 13:42:14 +0200 by phpDocumentor 1.3.0RC3
